# Fracture related infection complicating civilian ballistic wounds in the amasonian zone

**DOI:** 10.1007/s10096-025-05203-8

**Published:** 2025-07-05

**Authors:** Karamba Sylla, Xinggang Lu, Adrien Mons, Vincent Sainterose, Paul Le Turnier, Loic Epelboin, Felix Djossou, Arsène A. Kpangon, Philippe Abboud, Fouzia Sariak, Richard Naldjinan, Renaud Scussel, Morgane Bourne, Fredegonde About, Olivier Lesens

**Affiliations:** 1 Department of Infectious and Tropical Diseases, Cayenne Hospital, Centre Hospitalo Universitaire de Guyane, Cayenne, 97300 French Guiana; 2 Orthopaedic Department, Cayenne Hospital, Cayenne, French Guiana; 3 Bacteriology Department, Cayenne Hospital, Centre Hospitalo Universitaire de Guyane, Cayenne, French Guiana; 4 West Indies-Guyana Clinical Investigation Center, Inserm CIC 1424, Cayenne Hospital, Cayenne, French Guiana

**Keywords:** Osteoarticular infection, Ballistic wound, French Guiana

## Abstract

**Introduction:**

Fracture-Related Infections (FRI) are one of the most common complications of ballistic wounds sustained in military operations. There is very little data on ballistic wounds in civilians, especially FRIs. There is no data at all on FRIs in French Guiana, which has one of the highest rates of delinquency and gun ownership in France.

**Patients and methods:**

We conducted a retrospective study, including patients admitted to Cayenne Hospital with civilian ballistic wounds. Patients with incomplete records were excluded. The following data were collected: wound location, infectious complications and microbiological documentation. Chi-squared tests were used for categorical variables, a Student’s t-tests for continuous variables and a logistic regression for multivariate analysis.

**Results:**

Of the 196 patients included in the study, the overall reported infection rate was 14%. FRIs were the most frequent infectious complication, occurring in 15% (14 out of 95 fractures caused by ballistic wounds). Of these, 53 were open fractures, 27 of which were classified as Gustilo ≥ 2. Six (43%) of the 14 FRIs were monomicrobial, including four methicillin-susceptible *S. aureus*. Eight (57%) were polymicrobial, seven of which had at least one Gram-negative bacillus in culture. Sixteen Gram-negative bacilli, including ten group 3 Enterobacteriaceae, were isolated from eight patients. The presence of an external fixator (adjusted odds ratio (aOR) [95% confidence interval (CI)]: 28.9 [5.9-139.9], *p* < 0.001) and comorbidity (diabetes, steroids or HIV) (aOR [95% CI]: 37.5 [4.5-308.4], *p* = 0.001) were significantly associated with FRI occurrence in the multivariate analysis.

**Conclusion:**

FRIs are one of the most common complications from civilian ballistic wounds in French Guiana. FRIs are often polymicrobial, with a high prevalence of Gram-negative bacteria. FRIs are favored by comorbidities and the presence of an external fixator.

**Supplementary Information:**

The online version contains supplementary material available at 10.1007/s10096-025-05203-8.

## Introduction

The number of gunshot wounds is increasing worldwide [1]. South American countries have the highest rates of firearm-related violence. Venezuela, Brazil and Colombia have rates of 53.7, 25.2 and 23.7 firearm homicides per 100,000 inhabitants, respectively whereas the rate is 10.2 in South Africa and 2.8 in the USA [1]. From 2009 to 2017, the majority of non-fatal and overall firearm injuries in the USA were due to assaults or accidents, accounting for 85,694 emergency room visits during this period [2]. Although firearm-related mortality is declining in France [3], French Guiana remains one of the French territories with the highest crime rates and number of firearms [4]. French Guiana is a French territory in South America, bordering Brazil and Suriname. 95% of its territory is covered by the Amazon rainforest. Its public health system comprises three coastal hospitals and 17 Delocalized Prevention and Care Centers (DPCCs). Emergency transfers from a DPCC to a hospital are carried out by helicopter. Between 2021 and 2023, French Guiana’s homicide rate was 15.7 homicides per 100,000 inhabitants, compared to 1.3 in mainland France [5]. Between 2016 and 2019, around 290 cases of firearm injuries were recorded and treated at the Cayenne hospital, resulting in 21 deaths [4].

Contrary to the belief that the bullet “sterilizes” the ballistic trajectory, infection contributes to the prognosis of firearm injuries [6]. In fact, bullets tend to transfer bacteria and debris into the wound, and may fragment in the tissue [7]. A distinction is traditionally made between ballistic wounds caused by high-velocity weapons, which are generally associated with armed conflict, and those caused by low-velocity weapons, which are generally associated with civilian use [8]. War wounds are generally highly contaminated and more prone to infection [9]. The frequency of infections associated with gunshot wounds can vary considerably depending on a number of factors, including the context (military or civilian), injury type, medical care received and environmental conditions. Nevertheless, data on Fracture Related Infections (FRIs) complicating civilian and even military ballistic injuries are scarce (see Table 1) [9–13].

**Table 1:** Incidence, risk factors and infectious agents of FRIs^a^ caused by military and civilian ballistic wounds in the literature

Authors	Population	FRI^a^ rate	Risk factors for infection identified	Microbiology of FRIs^a^
Creusefond C et al. 2019, [10] (unpublished data)	265 civilian patients	22%	Accidental bullet, pellet-type projectiles, damage to arm or ankle, bone, vascular-nervous, musculotendinous or soft tissue damage, Gustilo IIIB and IIIC fractures	*Staphylococcus* sp. (44%), *Enterococcus* sp. (20%), streptococci (7%), anaerobes (7%) and fungus (12%)
Ghali AN et al. 2023 [11]	347 civilian patients	9.3%	Fracture of the lower limb, comminuted nature of fractures	*S. aureus* including 5 *MRSA* *Enterobacter* sp., *Citrobacter* sp., *Klebsiella* sp.
Burns TC et al. 2012 [12]	27 civilian patients	3.7%	Severe soft tissue damage Gustilo IIIB open fracture	*Enterobacter cloacae*
Penn-Barwell JG et al. 2016 [9]	97 military patients	23%	Significant bone loss	*Staphylococcus aureus* (*n* = *13)*,* Acinetobacter* (*n* = *3)*,* Pseudomonas sp.* (*n* = *2)*,* Staphylococcus Coag. Neg* (*n* = *1)*,* Enterobacter Sp* (*n* = *2)*
Burns TC et al. 2012 [12]	192 military patients	27%	NR	Gram-negative bacilli in 93% of cases
Brown KV et al. 2010 [13]	84 military patients	24%	Use of tourniquets in the field, antibiotics during evacuation and in the operating room, fasciotomy	Early infection: *S. aureus* Late infection: *Acinetobacter* sp.

^a^ FRI: Fracture related infection

The initial surgical management of gunshot wounds involves debriding necrotic or contaminated tissue. More extensive debridement is required in cases involving residual fragmented projectile or comminuted fracture [8]. In 1998, Ganocy and Lindsey proposed a classification system that takes into account the location of the projectile, the type of fracture and the level of contamination in order to determine the necessary surgical intervention (ranging from no surgery, to irrigation lavage with the extraction of bone fragments and/or the bullet and its residues) [14]. The surgical procedure is usually accompanied by antibiotic prophylaxis, which may continue as curative antibiotic therapy after the surgical procedure, depending on the severity of the wound and underlying fracture. The SFAR (Société Française d’Anesthésie et de Réanimation, French Society of Anaesthesia and Intensive Care) does not address gunshot wounds in its recommendations [15]. For open fractures, the SFAR recommends antibiotic prophylaxis with amoxicillin-clavulanic acid for Gustilo types 2 or 3 fractures. This involves 2 g intravenously, followed by 1 g every two hours until the end of surgery, starting within three hours of the fracture. However, the SFAR specifies that “curative antibiotic therapy should be considered beyond the operating room” for this type of open fractures [15]. Overall, in the absence of recommendations, there is considerable variation in the management of gunshot wounds between surgeons and institutions [16].

No study has investigated the infectious complications of ballistic wounds, particularly in cases of osteoarticular damage, in French Guiana or more generally in the Amazon Zone. The first objective of our study was to describe the clinical and microbiological characteristics of FRIs following ballistic trauma in civilians in French Guiana. Secondly, we sought to identify the factors associated with the occurrence of FRIs in these patients.

## Patients and methods

We conducted a retrospective observational study of the files of patients hospitalized for civilian ballistic wounds at Cayenne Hospital (French Guiana) between 08/15/2018 and 07/28/2023. The study was reported in accordance with the Strengthening the Reporting of Observational Studies in Epidemiology (STROBE) guidelines. Eligible patients were screened for ballistic wounds using the Cayenne hospital information system PMSI (information systems medicalization program). Data were anonymously collected from the electronic medical record. The following data were noted: Socio-demographic characteristics (age, sex, municipality of residence), anamnestic and clinical data (circumstances of wound occurrence, location of the wound, number of bullet impacts, fracture characteristics, number of organs affected, nature of the initial lesions: compartment syndrome, vascular and nervous involvement) and trauma-related infection microbiology data (cultures of deep sampling, joint fluid, bone biopsies and tissue samples) were noted. Open fractures were classified according to the Gustilo scale [17]. Infectious complications that were directly related to the ballistic wound were assessed through file review. Infections with no direct relation to ballistic injury such as catheter-related bacteremia, ventilator-acquired pneumonia or catheter-related urinary tract infections were not considered in this study. Patients with ballistic wounds affecting a bone or joint were assessed in this database to determine the frequency and nature of FRI in this subpopulation, as well as the risk factors for its occurrence. A diagnosis of FRI was considered if at least one of the four confirmatory criteria of the consensus FRI definition was met [18]: (1) Fistula, sinus, or wound breakdown. (2) Purulent drainage from the wound or presence of pus during surgery. (3) Identification of phenotypically indistinguishable pathogens by culture from at least two separate deep tissue or implant specimens. (4) The presence of microorganisms in deep tissue samples taken during an operative procedure, confirmed by histopathological examination.

### Microbiological samples

The processing of the samples was standardized. High-quality, deep surgical samples were inoculated onto four types of agar media (including both selective and non-selective types) and incubated under aerobic conditions enriched with 5% CO_2_. Two additional types of agar media were incubated under anaerobic conditions, and an enrichment broth was also used. For polymicrobial samples, all microorganisms were identified, and antibiotic susceptibility was determined for each bacterium.

### Ethics

The study was in accordance with the French MR004 reference methodology. MR-004 is a rule adopted by the French data protection authority (“CNIL”) for the processing of personal data within the context of an observational study. Because this retrospective study used anonymized healthcare data, the necessity for patient consent was obviated in accordance with French legislation. In accordance with the European regulation on data protection, the study was registered in the hospital’s register of processing activities by the person responsible for data protection, and collective information was made available to patients by means of a poster in the department, allowing them to express their refusal to participate if necessary.

### Statistical analysis

Patient characteristics were expressed as numbers and percentages for categorical variables and as mean and standard deviation for quantitative variables. Comparisons between infected versus non-infected groups among patients with ballistic bone or joint involvement were compared, for quantitative variables, by the Student t-test or the Mann-Whitney test if the conditions for applying the t-test were not met. Comparisons between groups for categorical parameters were performed with the Chi2 test or, if necessary, the Fisher exact test. In a second step, a multivariate approach by logistic regression model (for the binary dependent variable bone or joint infection yes/no) was proposed by considering the adjustment variables with regard to the results of the univariate analysis and their clinical relevance. Analyses were performed with Stata software (version 12, Stata Corp, College Station, US). All tests were bilateral with a type 1 error set at 5%.

## Results

### Characteristics of patients with at least one ballistic wound (Fig. 1)

**Fig. 1:**
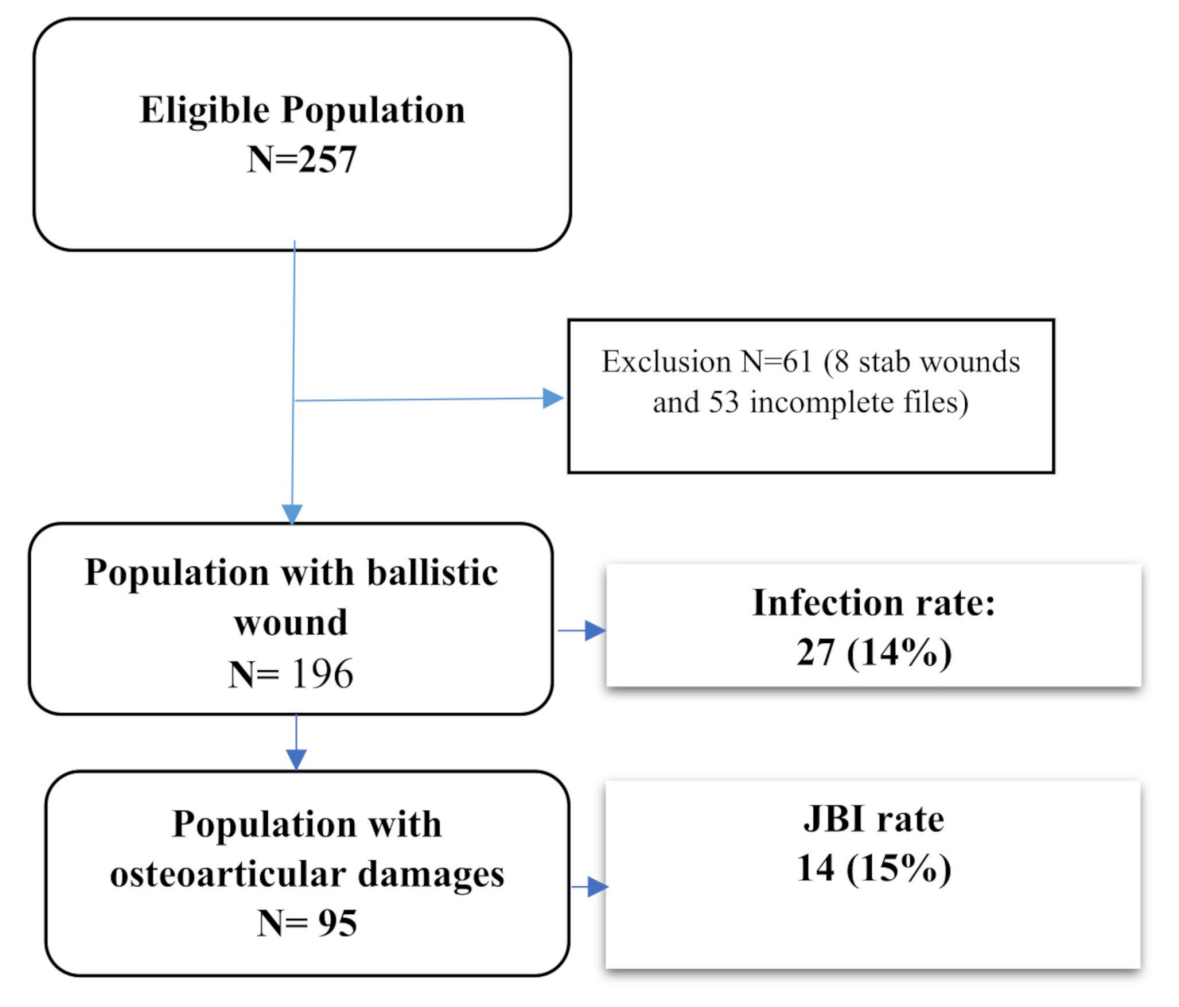
Flow chart of the study

The study population comprised 196 patients, 186 (95%) of whom were male. The mean age was 31 ± 11 years [range: 11–63]. Of the comorbidities, seven patients had well-controlled diabetes, one had a solid tumor, and two had HIV infection that was immunologically and virologically well controlled. The origin of the ballistic wound was caused by hetero-aggression in 177 (91%) patients, a hunting and/or weapon handling accident in 13 (7%) and a suicide attempt in one patient. The circumstances were not reported for five patients. Twenty-seven (14%) patients presented with an infectious complication, including fourteen with FRI, four with an abdominopelvic infection, six with a skin and soft tissue infection and two with an intrathoracic infection.

### Characteristics of patients with osteoarticular damage following a ballistic wound

Of the 196 patients, 95 (48.5%) had at least one fracture affecting the lower limb; 30 (15.3%) had a fracture affecting the upper limb; 23 (11.7%) had a fracture affecting the pelvis; 5 (2%) had a fracture affecting the spine; 12 (6%) had a fracture affecting a bone of the ENT (Ear-Nose-Throat); and nine (11%) had a fracture affecting other bones or joints. Of the 53 fractures localized to the limbs, 26 (49%) were classified as Gustilo I, 17 (32%) as Gustilo II, 4 (8%) as Gustilo IIIA, 5 (9%) as Gustilo IIIB and 1 (2%) as Gustilo IIIC. All patients with a fracture complicating a gunshot wound received antibiotic prophylaxis with amoxicillin-clavulanic acid, which was administered at the beginning of the interventional procedure and continued for 48 h afterwards.

Fourteen (15%) of the 95 patients with bone fractures developed FRI: eight patients had osteitis on external fixators (six tibiae and two femurs); two had contiguous osteitis (one tibia and one femur); one had contiguous knee arthritis; and two had osteitis associated with nail implants (one tibial and one femoral). Seven patients required more than one operation, including five for FRI.

The characteristics of these patients who developed a post-ballistic FRI were compared with those who did not (see Table 2). The occurrence of FRI in patients with civil ballistic bone fractures was associated with comorbidity (diabetes, steroids or HIV) and several correlated variables including severe soft tissue damage, Gustilo ≥ 2, use of an external fixator, tibial fracture and lower limb involvement (Table 2). Use of an external fixator (Adjusted Odds Ratio (aOR) [95% Confidence Interval (CI)]: 28.9 [5.9-139.9], *p* < 0.001) and comorbidity (aOR [95% CI]: 37.5 [4.5-308.4], *p* = 0.001) were significantly associated with the occurrence of FRI in multivariate analysis.

**Table 2:** Factors associated with the occurrence of FRIs in patients with ballistic bone fracture (*n* = 95) in univariate analysis

Variables	FRI^a^ *n* = 14	No FRI^a^ *n* = 81	Total *n* = 95	*p*
Mean age ± SD^b^	35.3 ± 2.9	31.2 ± 0.8	31.8 ± 1.2	0.219
**Comorbidity**^**c**^, **n (%)**	**4 (28.6)**	**2 (2)**	**6 (6.3)**	**0.004**
Upper limb, n (%)	1 (7.1)	26 (32.1)	27 (28.4)	0.056
**Lower limb**, n (%)	**13 (92.9)**	**25 (40)**	**38 (40)**	**< 0.001**
Tibial fracture	6 (42.9)	8 (9.9)	14 (17.7)	0.001
Unique ballistic impact, n (%)	11 (78.6)	54 (66.7)	65 (68.4)	0.376
Muscle damage, n (%)	2 (14.3)	8 (9.9)	10 (10.5)	0.620
Vascular lesion, n (%)	2 (14.3)	4 (4.9)	6 (6.3)	0.184
**Severe soft tissue damage**, n (%)	**8 (57.1)**	**11 (13.6)**	**19 (20)**	**< 0.001**
Lodge syndrome, n (%)	2 (14.3)	4 (4.9)	6 (6.3)	0.184
Gustilo ≥ 2***, n (%)	**8 (57.1)**	**22 (27.2)**	**30(31.6)**	**0.026**
Osteosynthesis material				
Nail implant	2 (14.3)	8(9.9)	10 (10.5)	0.620
Screw/plate	1 (7.1)	6 (7.4)	7 (7.4)	0.972
** External fixator** ** Temporary external fixator**	**8 (57.1)** **1 (7.1)**	**6 (7.4)** **0**	**14 (14.7)** **1 (1%)**	**< 0.001** **0.13**

^a^FRI: Fracture Related infection; ^b^SD: standard deviation; ^c^comorbidity: diabetes, steroids or HIV); ^d^Gustilo-Anderson classification for soft tissue wounding of open fractures [17]

*This table describes the risk factors associated with the occurrence of osteoarticular infections secondary to ballistic wounds in French Guiana

**Fig. 2:**
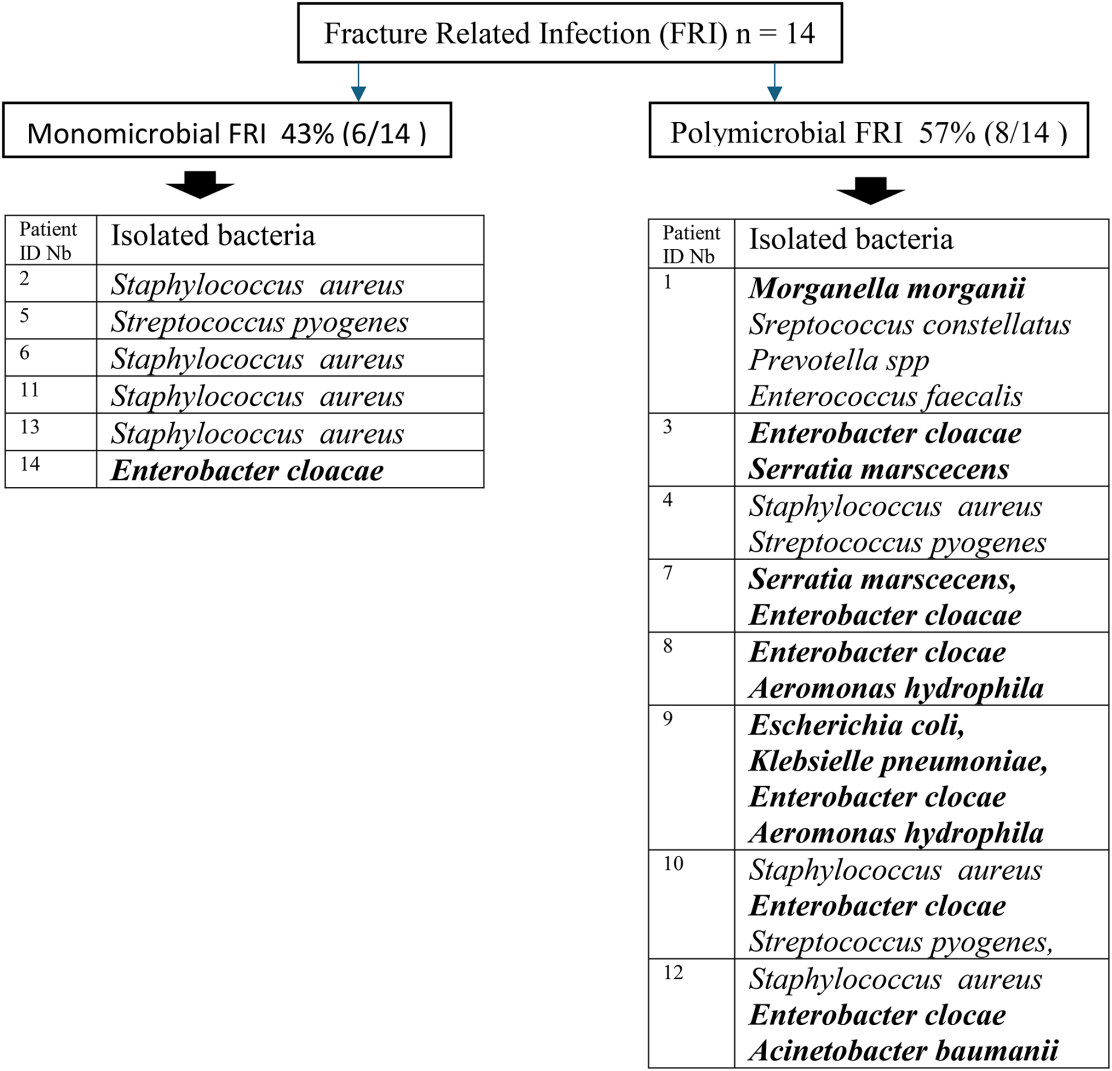
Microorganisms isolated in culture in 14 FRIs. Gram-negative bacilli are shown in bold. Patient ID NB : Patient IDentification Number

Of the 14 FRIs, six (43%) were monomicrobial, including four methicillin-susceptible *S. aureus* (MSSA) (see Fig. 2). Eight (57%) were polymicrobial, seven of which had at least one Gram-negative bacillus in culture. Sixteen Gram-negative bacilli, including ten Group 3 Enterobacteriaceae, were isolated from eight patients. Twelve gram-positive cocci, including six methicillin-susceptible *S. aureus* and four *Streptococci*, were found in nine patients.

## Discussion

In this series of 196 consecutive cases of ballistic wounds in French Guyana, the overall infection rate was 14%. FRI were the most frequent infectious complication, occurring in 15% of the 95 fractures caused by ballistic wounds. Of these, 53 were open fractures, 27 of which were classified as Gustilo ≥ 2. For these fractures, the preferred treatment was osteosynthesis using an external fixator. In multivariate analysis, comorbidity and the presence of an external fixator — which is frequently used in open fractures — were significantly associated with the occurrence of FRI. The micro-organisms involved were mainly Gram-negative bacilli, notably group 3 Enterobacteriaceae. There were also two cases of infection involving *Aeromonas hydrophila*, a natural resident of the aquatic environment [19]. This study focuses on two aspects that have received limited attention in existing literature. Firstly, it considers civilian ballistic wounds, which differ from military ballistic wounds in several ways. Military ballistic wounds are characterized by extensive soft tissue damage caused by the high-energy weapons used in combat. Those associated with combat have a high incidence of infection, often involving multidrug-resistant Gram-negative bacteria [12]. In contrast, civilian ballistic fractures are often considered low-velocity traumas with a lower infection rate, with staphylococci being the main causative microorganism [10]. Secondly, the specific geographical location of French Guiana in the Amazon region results in a different profile of microorganisms compared to temperate zones. The results of our study provide an opportunity to improve our understanding of the specific features of these infections in the Amazon region and, potentially, in other tropical zones. In French Guiana, a large number of ballistic wounds occur in areas that are not easily accessible, and in a context of illegal gold panning. Open fractures are rarely treated within 6 h, as is usually recommended (in 2023, no open fracture operated on in the Cayenne orthopedics department could be operated on within 6 h, personal data). Nevertheless, this 6-hour delay appears to be dogmatic and does not stand up to analysis in the literature [20]. The time window to manage an open fracture and limit the best the risk of infection is not known, but could be around 24 h [20]. This may partly explain why the rate of FRIs remains relatively limited in our series despite a delayed care. Similarly, the humid climatic conditions and high probability of telluric and aquatic contamination specific to Amazonia do not seem to influence the incidence of infectious complications in these patients.

Environmental characteristics seem to influence the nature of the micro-organisms present in these infections. While Gram-positive cocci, and in particular *Staphylococcus aureus*, seem to dominate the causes of post-bullet osteoarticular infections in the literature, Gram-negative bacilli are in the majority, with an over-representation of group 3 Enterobacteriaceae and the presence of *Aeromonas* sp. This characteristic is noted for skin abscesses occurring in Amazonian zones [21]. The lower limbs are particularly exposed to telluric soiling, and are readily exposed to this type of micro-organism. This observation may have two consequences. Firstly, it may affect the choice of antibiotic prophylaxis. The amoxicillin-clavulanic acid recommended by the SFAR for open fractures is active against *Clostridium perfringens*, *Streptococcus pyogenes* and methicillin-susceptible *S. aureus* [15]. This antibiotic prophylaxis may not provide adequate coverage for group 3 Enterobacteriaceae or *Aeromonas* sp in this specific epidemiological context. An alternative that could be suggested would be the use of trimethoprim-sulfamethoxazole as a replacement for amoxicillin-clavulanic acid, at least for Gustilo II fractures of the lower limb. The advantage of this alternative would be to avoid the proliferation of microorganisms after surgery due to inadequate antibiotic prophylaxis, while using an antibiotic that selects relatively little resistance. The value of antibiotic prophylaxis between the time of occurrence of the ballistic wound and the time of surgery remains to be demonstrated, but is probably worthwhile, particularly when the time to surgery is long.

The second consequence concerns empiric antibiotic therapy for these post-ballistic wound infections. Monotherapy with cefepime or piperacillin-tazobactam could be proposed. Piperacillin-tazobactam has the advantage of being effective against enterococci (found in one case in our study), but its efficacy against *Aeromonas* sp is questionable. As no MRSA was found, the value of adding probabilistic antibiotic therapy active against this bacterium (daptomycin, linezolid, vancomycin) is also debatable. Once the results of microbiological sampling are known, oral treatment can be rapidly initiated, including, in the case of Gram-negative bacilli, a fluoroquinolone or cotrimoxazole.

The main goals of treatment are to achieve fracture consolidation with a satisfactory functional outcome, to eradicate infection or at least suppress it until consolidation and removal of the material, and finally to restore soft tissue integrity [22]. Surgically, the initial intervention to reduce the fracture most often uses either an intramedullary nail or an external fixator. The use of an intramedullary nail is reportedly not associated with a higher incidence of infection, but with a better functional prognosis and improved quality of life [23]. Nevertheless, in the event of infection of the osteosynthesis material, the presence of an intramedullary nail practically obliges us to opt for a one- or two-stage material change or to replace the implant with an external fixator rather than opt for conservative treatment (Debridement Antibiotics and Implant Retention DAIR). The duration of antibiotic treatment has not been codified and is not the subject of any recommendation. In the absence of implant, a 6-week course of antibiotics should be sufficient. In the presence of implant, which represents the majority of cases, a 12-week course may be considered, as recommended in the treatment of prosthesis infections. Removal of the material once consolidation has been achieved should be the rule, to avoid the risk of late infection.

This study has limitations. Its retrospective and observational nature may have adversely affected data quality with a risk of incomplete or inaccurate data. We took into account infections that occurred during the initial hospitalization or infections that led to re-hospitalization in our establishment. Some patients may have been lost to follow-up, notably illegal gold miners, and some late-onset infections may not have been taken into account. Furthermore, the size of our sample with FRI complicating a ballistic wound is modest and does not allow us to draw any conclusions regarding the management of these infections. Finally, the monocentric nature of the study means that the results are not necessarily representative of the Amazon region, particularly Brazil. The value of empirical antibiotic therapy that is better adapted to the microorganisms found in the Amazon zone, and more targeted at certain Gram-negative bacilli, notably group 3 Enterobacteriaceae and *Aeromonas sp*, needs to be confirmed in a prospective study.

## Conclusion

FRIs are one of the most common complications of civilian gunshot wounds in French Guiana. FRIs are often polymicrobial, with a high prevalence of Gram-negative bacteria. FRIs are favored by comorbidities and the presence of an external fixator. The value of empirical antibiotic therapy that is better adapted to the microorganisms found in the Amazon zone, and more targeted at certain Gram-negative bacilli, notably group 3 Enterobacteriaceae and *Aeromonas* sp, needs to be confirmed in a prospective study.

## Electronic supplementary material

Below is the link to the electronic supplementary material.

Supplementary Material 1

Supplementary Material 2

Supplementary Material 3

Supplementary Material 4

## Data Availability

No datasets were generated or analysed during the current study.
