# Supplementary material for: Fracture related infection complicating civilian ballistic wounds in the amasonian zone
Source: Eur J Clin Microbiol Infect Dis. 2025 Jul 5;44(10):2401–8. doi: 10.1007/s10096-025-05203-8 (PMC12484085; doi:10.1007/s10096-025-05203-8)
Supplement: Supplementary file 1 — Supplementary Material 1 [file 10096_2025_5203_MOESM1_ESM.docx]

**Table 1: Incidence, risk factors and infectious agents of BJIs caused by military and civilian ballistic wounds in the literature**

| Authors | Population | BJI rate | Risk factors for infection identified | Microbiology of BJIs |
| --- | --- | --- | --- | --- |
| Creusefond C et al. 2019,  (5) (unpublished data) | 265 civilian patients | 22% | Accidental bullet, pellet-type projectiles, damage to arm or ankle, bone, vascular-nervous, musculotendinous or soft tissue damage, Gustilo IIIB and IIIC fractures | Initial episode: *Staphylococcus* sp. (44%), *Enterococcus* sp. (20%), streptococci (7%), anaerobes (7%) and fungus (12%)  Recurrence: *Staphylococcus* sp. 38%, *Streptococcus* sp. 14%, *Enterococcus* sp. 10%, Gram-negative bacilli 38%. |
| Ghali AN et al 2023 (6) | 347 civilian patients | 9.3% | Fracture of the lower limb, comminuted nature of fractures | *S. aureus* including 5 *MRSA*  *Enterobacter* sp., *Citrobacter* sp., *Klebsiella* sp. |
| Burns TC et al 2012 (7) | 27 civilian patients | 3.7% | Severe soft tissue damage  Gustilo IIIB open fracture | *Enterobacter cloacae* |
| Penn-Barwell JG et al. 2016 (4) | 97 military patients | 23% | Significant bone loss | *Staphylococcus aureus (n=13), Acinetobacter (n=3), Pseudomonas* sp. *(n=2), Staphylococcus Coag. Neg (n=1), Enterobacter Sp (n= 2)* |
| Burns TC et al. 2012 (7) | 192 military patients | 27% | NR | Gram-negative bacilli in 93% of cases |
| Brown KV et al. 2010 (8) | 84 military patients | 24% | Use of tourniquets in the field, antibiotics during evacuation and in the operating room, fasciotomy | Early infection: *S. aureus*  Late infection: *Acinetobacter* sp. |

*This table describes the incidences, risk factors and microorganisms isolated in osteoarticular infections secondary to ballistic wounds in the literature.
