# Supplementary material for: Fracture related infection complicating civilian ballistic wounds in the amasonian zone
Source: Eur J Clin Microbiol Infect Dis. 2025 Jul 5;44(10):2401–8. doi: 10.1007/s10096-025-05203-8 (PMC12484085; doi:10.1007/s10096-025-05203-8)
Supplement: Supplementary file 2 — Supplementary Material 2 [file 10096_2025_5203_MOESM2_ESM.docx]

**Table 2: factors associated with the occurrence of BJIs in patients with ballistic bone fracture (n=95) in univariate analysis**

| Variables | BJI*  n=14 | No BJI*  n = 81 | Total  n=95 | p |
| --- | --- | --- | --- | --- |
| Mean age ± SD** | 35.3±2.9 | 31.2±0.8 | 31.8 ± 1.2 | 0.219 |
| Upper limb, n (%) | 1 (7.1) | 26 (32.1) | 27 (28.4) | 0.056 |
| **Lower limb**, n (%) | **13 (92.9)** | **25 (40)** | **38 (40)** | **<0.001** |
| Unique ballistic impact, n (%) | 11 (78.6) | 54 (66.7) | 65 (68.4) | 0.376 |
| Muscle damage, n (%) | 2 (14.3) | 8 (9.9) | 10 (10.5) | 0.620 |
| Vascular lesion, n (%) | 2 (14.3) | 4 (4.9) | 6 (6.3) | 0.184 |
| **Severe soft tissue damage**, n (%) | **8 (57.1)** | **11 (13.6)** | **19 (20)** | **<0.001** |
| Lodge syndrome, n (%) | 2 (14.3) | 4 (4.9) | 6 (6.3) | 0.184 |
| **Gustilo ≥ 2**, n (%) | **8 (57.1)** | **22 (27.2)** | **30(31.6)** | **0.026** |
| Osteosynthesis material |  |  |  |  |
| Nail implant | 2 (14.3) | 8(9.9) | 10 (10.5) | 0.620 |
| Screw/plate | 1 (7.1) | 6 (7.4) | 7 (7.4) | 0.972 |
| **External fixator** | **8 (57.1)** | **6 (7.4)** | **14 (14.7)** | **<0.001** |

*BJI: bone and joint infection; **SD: standard deviation;

*This table describes the risk factors associated with the occurrence of osteoarticular infections secondary to ballistic wounds in French Guiana.
