# Supplementary material for: Fracture related infection complicating civilian ballistic wounds in the amasonian zone
Source: Eur J Clin Microbiol Infect Dis. 2025 Jul 5;44(10):2401–8. doi: 10.1007/s10096-025-05203-8 (PMC12484085; doi:10.1007/s10096-025-05203-8)
Supplement: Supplementary file 3 — Supplementary Material 3 [file 10096_2025_5203_MOESM3_ESM.docx]

**Figure 1: flow chart of the study**

***Flow chart of patients hospitalized at the Cayenne Hospital over five years for civilian ballistic wounds complicated by osteoarticular infection**

**Eligible Population**

**N=257**

**Population with osteoarticular damages**

**N= 95**

**Infection rate:**

**27 (14%)**

Exclusion N=61 (8 stab wounds and 53 incomplete files)

**Population with ballistic wound**

**N=** 196

**JBI rate**

**14 (15%)**
