# Supplementary material for: Fracture related infection complicating civilian ballistic wounds in the amasonian zone
Source: Eur J Clin Microbiol Infect Dis. 2025 Jul 5;44(10):2401–8. doi: 10.1007/s10096-025-05203-8 (PMC12484085; doi:10.1007/s10096-025-05203-8)
Supplement: Supplementary file 4 — Supplementary Material 4 [file 10096_2025_5203_MOESM4_ESM.docx]

*This figure shows the germs isolated in the deep samples of the fourteen patients who developed an osteoarticular infection following a gunshot wound at the Guiana Hospital.
